# Epithelial–mesenchymal transition in tumor metastasis

**DOI:** 10.1002/1878-0261.12017

**Published:** 2016-12-09

**Authors:** Kay T. Yeung, Jing Yang

**Affiliations:** ^1^ Department of Pharmacology University of California, San Diego La Jolla CA USA; ^2^ Department of Medicine University of California, San Diego La Jolla CA USA; ^3^ Moores Cancer Center University of California, San Diego La Jolla CA USA; ^4^ Department of Pediatrics University of California, San Diego La Jolla CA USA

**Keywords:** cancer, circulating tumor cells, epithelial–mesenchymal transition, invasion, metastasis, progression

## Abstract

The epithelial–mesenchymal transition (EMT) is a developmental program that enables stationary epithelial cells to gain the ability to migrate and invade as single cells. Tumor cells reactivate EMT to acquire molecular alterations that enable the partial loss of epithelial features and partial gain of a mesenchymal phenotype. Our understanding of the contribution of EMT to tumor invasion, migration, and metastatic outgrowth has evolved over the past decade. In this review, we provide a summary of both historic and recent studies on the role of EMT in the metastatic cascade from various experimental systems, including cancer cell lines, genetic mouse tumor models, and clinical human breast cancer tissues.

**Abbreviations**
CTC: circulating tumor cell
DMBA: 7,12‐dimethylbenz[a]anthracene
ECM: extracellular matrix
ELF5: E74‐like ETS transcription factor 5
EMT: epithelial–mesenchymal transition
EMT‐TF: epithelial–mesenchymal transition transcription factor
ERK2: mitogen‐activated protein kinase 2
FSP1: fibroblast‐specific protein 1
GFP: green fluorescent protein
GFRHL2: grainyhead‐like transcription factor 2
GSK3β: glycogen synthesis kinase 3 beta
HIF‐1α: hypoxia‐induced factor 1 alpha
IL‐6: interleukin‐6
JAM1/A: junctional adhesion molecular 1/A
MET: mesenchymal–epithelial transition
MMP: metalloproteinase
MMTV: mouse mammary tumor virus
NOTCH: neurogenic locus notch homolog protein
OVOL: ovo‐like zinc finger
PDX1: pancreatic and duodenal homeobox 1
PyMT: polyoma middle T oncoprotein
ROCK: Rho‐associated coiled‐coil containing protein kinase
SNAIL1: Snail family zinc finger 1
STAT: signal transducer and activator of transcription
TGF‐β: tumor growth factor beta
TPA: 12‐O‐tetradecanoylphorbol‐13‐acetate
TWIST1: Twist family BHLH transcription factor 1
WNT: wingless
YFP: yellow fluorescent protein
ZEB: zinc finger E‐box binding homeobox
ZO‐1: zonula occludens 1

## 1 Introduction

The metastatic cascade is a complex, highly inefficient, but deadly process that has been under intense investigation in hopes to eliminate distant spread and reduce cancer‐associated mortality. During carcinoma metastasis, stationary epithelium‐derived tumor cells must first become migratory and invasive in order to disseminate from the primary tumors and enter into the circulation. Upon surviving the transport, some circulating tumor cells (CTCs) manage to extravasate out of the vasculature and initiate distant metastasis colonization. At distant sites, disseminated tumor cells need to adapt to the new microenvironment and create a metastatic supportive niche, and switch from the migratory mode to proliferation mode to generate macrometastases.

A key event in promoting stationary tumor cells to migrate and invade is the epithelial–mesenchymal transition (EMT) program. A large body of work in the past 15 years has brought EMT to the forefront of metastasis research. This review aimed to summarize and highlight key evidence on the role of EMT in the metastatic cascade from studies using human cancer cell lines, cancer mouse models, and clinical human cancer tissues. The associated challenges and caveats with each approach will be presented to address the ongoing controversy on EMT in metastasis.

## 2 Overview of EMT

The EMT program was originally described as an integral part of morphogenesis in embryonic development, and later was observed in several pathogenesis events, including wound healing, fibrosis, and cancer metastasis. The hallmarks of the EMT program are loss of adherence junctions and apical–basal polarity, acquisition of mesenchymal phenotype, and gain of motility and invasion (Nieto, 2013). Epithelial cells are characterized by intact cell–cell interactions through adhesion molecules such as E‐cadherin and cytokeratin within tight junctions, adherens junctions, desmosomes, and gap junctions. Apical–basal polarity is also a key epithelial feature. In response to various extracellular cell‐ and tissue‐specific EMT‐inducing signals, epithelial cells upregulate a group of EMT‐inducing transcription factors to orchestrate all the morphological, cellular, and molecular changes during EMT. As this review focuses on EMT in tumor metastasis, we briefly summarize the key molecular regulators of EMT in cancer.

Various developmental signaling pathways such as TGF‐β, WNT, NOTCH, and growth factor receptor tyrosine kinases have been implicated in inducing EMT under certain physiological conditions. Tumor growth factor beta (TGF‐β), a cytokine secreted by tumor cells and stromal fibroblasts in the tumor microenvironment, is considered a primary inducer of EMT (Katsuno *et al*., 2013). Other signal transduction pathways implicated in EMT induction are inflammatory cytokines such as TNF‐α via NF‐κB (Wu *et al*., 2009) and IL‐6/STAT pathway (Lo *et al*., 2007); hypoxia via HIF‐1α (Yang *et al*., 2008); and extracellular matrix (ECM) stiffness (Wei *et al*., 2015).

In response to various EMT‐inducing cues and their downstream signaling pathways, EMT transcription factors (EMT‐TFs) are activated to orchestrate the EMT program. The main EMT‐TFs include the SNAIL family zinc finger transcription factors (SNAIL1 and SNAIL2), the TWIST family basic helix–loop–helix transcription factors (TWIST1 and TWIST2), and the zinc finger E‐box binding homeobox proteins (ZEB1 and ZEB2). SNAIL and ZEB transcription factor superfamilies are capable of binding to the E‐box sequences in the promoters of E‐cadherin to repress transcription (Batlle *et al*., 2000; Comijn *et al*., 2001). Members of the SNAIL and ZEB families have also been shown to downregulate tight junction proteins including occludin, claudins, ZO‐1, and connexins JAM1/A (Bax *et al*., 2011; Ikenouchi *et al*., 2003; Martínez‐Estrada *et al*., 2006; Ohkubo and Ozawa, 2004; Vandewalle *et al*., 2005). On the other hand, TWIST family members repress E‐cadherin expression through induction of SNAIL transcription factors in both Drosophila and humans (Fang *et al*., 2011; Perez‐Moreno *et al*., 2001; Yang *et al*., 2004). Furthermore, TWIST1 has been found to induce invadopodia‐mediated matrix degradation, thus promoting basement membrane degradation during EMT (Wei *et al*., 2015).

Epithelial–mesenchymal transition transcription factor activation leads to downregulation of genes encoding epithelial junction proteins, thus disassembling adherens junctions, desmosomes, and tight junctions and loss of apical–basal polarity (Qin *et al*., 2005). Because these junctions not only provide epithelial structure support but also regulate a number of signaling pathways via their associated proteins, changes in epithelial junctions impinge on a number of downstream pathways that might further promote EMT and invasion. For example, released beta‐catenin from adherences junctions could potentially enter the nucleus to drive WNT‐target gene expression to potentiate EMT (Howard *et al*., 2011).

At the same time, expression of mesenchymal genes such as N‐cadherin, fibronectin, vimentin is upregulated (Porta‐de‐la‐Riva *et al*., 2011). In particular, the expression of a number of nonepithelial cadherin and cell surface proteins critical for cell migration is increased. As the cytokeratin network anchored to desmosomes in epithelial cells is being deconstructed, upregulation of vimentin leads to reorganization of the cortical actin cytoskeleton into cytoplasmic and basal network of intermediate filaments (Beaty and Condeelis, 2014). This transition to mesenchymal state facilitates cell motility and the formation of new membrane protrusions such as invadopodia for matrix degradation. Actin stress fibers also form and increase cell contractility. Finally, increased cell protrusions and expression of metalloproteinases (MMPs) result in ECM degradation, cell migration, and invasive behavior (Eckert *et al*., 2011; Leong *et al*., 2014).

The EMT program is also controlled by multiple regulatory pathways such as miRNA (miR‐200 and miR‐34), long noncoding RNAs, and post‐translational modification (Xu *et al*., 2016). Negative regulators of EMT‐TF expression, such as OVOL, GRHL2, and ELF5, directly repress transcription of ZEB1 and SNAIL2, respectively (Chakrabarti *et al*., 2012; Cieply *et al*., 2012). miR‐200 family members have been demonstrated to form a negative regulatory loop with ZEB1 and ZEB2 and are able to modulate the plasticity between epithelial and mesenchymal states (Burk *et al*., 2008; Gregory *et al*., 2008; Korpal *et al*., 2008; Park *et al*., 2008). Ubiquitin‐mediated proteasome degradation of SNAIL1 and TWIST1 after GSK3β or MAP kinase phosphorylation is another mechanism of EMT‐TF modulation (Hong *et al*., 2011; Zhou *et al*., 2004).

**Figure 1:**
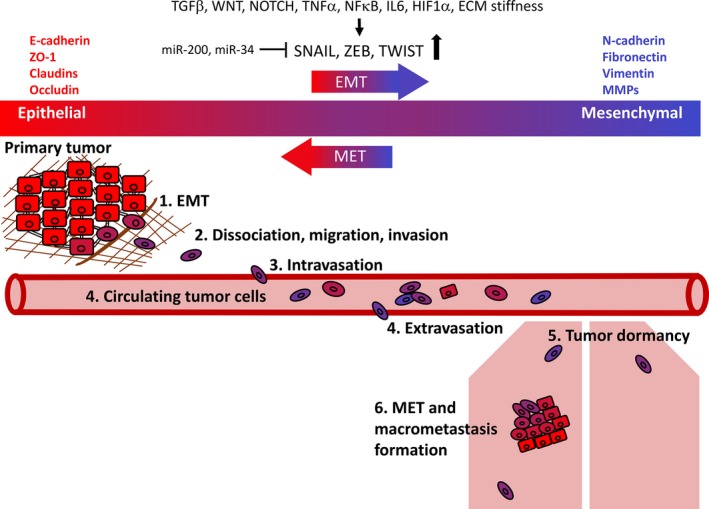
Primary epithelial tumor cells undergo epithelial–mesenchymal transition (EMT) to acquire the ability to disseminate from the original site and migrate into the surrounding stroma, and intravasate into circulation. A small number of circulating tumor cells and cell clusters survive in the vasculature and eventually extravasate into distant organs such as the lung. Disseminated tumor cells could remain in dormant state for a period of time before undergoing mesenchymal–epithelial transition (MET) to proliferate and form macrometastases.

In summary, EMT is a highly complex and dynamic process with numerous overlapping pathways that can simultaneously exert their regulatory arms to orchestrate downstream events (Fig. 1). Therefore, the EMT program is not simply a binary process, but rather a spectrum of reversible cell states in transition. Very often, epithelial cells could undergo a partial EMT and present with partial epithelial or mesenchymal states. This characteristic of EMT in cancer has important implications in its role in tumor metastasis, which will be discussed in detail in the following sections.

## 3 EMT studies in tumor cell lines

The link between EMT and tumorigenesis was first noted in advanced epithelial cancer cell lines. Downregulation of E‐cadherin expression or function by antisense RNA or E‐cadherin antibody led to an increased invasion and adaption of a fibroblast morphology in breast, lung, bladder, pancreas epithelial tumor cell lines (Frixen *et al*., 1991; Vleminckx *et al*., 1991). In addition, re‐expression of E‐cadherin in invasive carcinoma cell lines reduced their ability to invade *in vitro* (Frixen and Nagamine, 1993; Perl *et al*., 1998; Vleminckx *et al*., 1991). Subsequently, TGF‐β became known as a major EMT‐inducing cytokine when it was found to transform mouse mammary epithelial NMuMG cells with an epithelial morphology into an invasive fibroblastoid phenotype. The morphological changes were also coupled with changes in classic EMT markers, including a loss of E‐cadherin expression and increased expression of vimentin (Oft *et al*., 1996). Further studies show that TGF‐β is able to induce EMT in various cell lines such as Ras‐transformed mammary epithelial (EpRas) cells and mouse colon carcinoma hnPCC cells (Miettinen *et al*., 1994; Oft *et al*., 1998). Importantly, inhibition of the TGF‐β pathway with a dominant negative type II TGFβR, neutralizing TGF‐β antibodies, or soluble receptor variants in mesenchymal mouse colon carcinoma CT26 cell line was shown to decrease *in vitro* invasiveness and metastasis formation in tumor xenografts (Oft *et al*., 1998). These studies suggest a plausible link between EMT and metastasis. However, due to the numerous biological functions of TGF‐β, it is difficult to pinpoint in these studies that the key function of TGF‐β in metastasis is due to EMT.

In 2000, two research groups independently identified SNAIL1 as the EMT‐TF that directly binds to the E‐cadherin promoter and controls E‐cadherin gene expression. Overexpression of SNAIL1 in breast cancer cell lines led to the loss of E‐cadherin‐mediated cell–cell adhesion, a dramatic change from epithelial to mesenchymal spindle morphology, and an increase in migratory and invasive phenotype (Batlle *et al*., 2000; Cano *et al*., 2000). Subsequent studies showed that SNAIL1 was also able to induce EMT in various epithelial cancer cell lines and primary mammary tumor cells (Moody *et al*., 2005). In addition, SNAIL1 overexpression in primary Neu‐driven mammary tumor grafts led to an increased rate of tumor recurrence, after transplantation into nude mice (Moody *et al*., 2005). The direct link of EMT‐TF to tumor metastasis was established upon the discovery of another EMT‐TF, TWIST1 in tumor metastasis. TWIST1 was found to induce EMT via SNAIL2‐induced suppression of E‐cadherin (Casas *et al*., 2011) and to promote matrix degradation via induction of invadopodia (Eckert *et al*., 2011). Knockdown of TWIST1 expression in mammary carcinoma 4T1 cells suppressed the ability of 4T1 cells to metastasize from the mammary gland to the lung mice. Together, these data brought the EMT research into the forefront of the metastasis field.

Many follow‐up studies further strengthen the notion that EMT‐TFs are necessary for metastasis in cell lines. In head‐and‐neck cell lines, TWIST1 was found to be an essential downstream target of HIF‐1α and that knockdown of TWIST1 led to a decreased number of metastatic nodules after either tail vein injection or orthotopic transplantation of hypopharyngeal cancer cell line with overexpression of HIF‐1α (Yang *et al*., 2008). Interestingly, ECM stiffness, a mechanosensory signal, was shown to activate EMT via TWIST1 and lead to increased tumor invasion and metastasis (Wei *et al*., 2015). Activation of integrin‐dependent mechanotransduction pathway in response to stiffening of ECM was shown to induce nuclear translocation of TWIST1 by releasing TWIST1 from its cytoplasmic anchor G3BP2. In addition, nuclear translocation of TWIST2 promotes invasion and tumor metastasis in breast tumor xenograft model. On the other hand, stiffness also leads to stabilization and nuclear accumulation of SNAIL1 protein via Rho‐associated coiled‐coil containing protein kinase (ROCK) and ERK2 pathways in breast tumor cells and cancer‐associated fibroblasts (Zhang *et al*., 2016).

In summary, studies in various tumor cell lines established the first direct link between EMT and metastasis. Genetic manipulation and phenotype evaluation using 2D and 3D culture led to the identification of EMT regulators in invasion with confirmation of its role in metastasis in mouse tumor xenografts. Also importantly, a large body of mechanistic studies performed in tumor cell lines revealed a complex genetic network that regulates EMT and identified potential key therapeutic targets. However, studies of EMT in cancer cell lines also have its drawbacks. In most 2D culture systems, loss of epithelial polarity cannot be studied due to the lack of apical–basal polarity. Although 3D culture systems can partially recapitulate tumor tissue architecture, the time‐dependent tumor progression cannot be easily recapitulated *in vitro*. Implantation of breast cancer cell lines in mice has been effective in providing the functional validation of EMT in driving metastasis. However, most tumor cell lines used are fixed in an epithelial or mesenchymal state, partially due to *in vitro* culture condition; thus, the plasticity of EMT program during tumor metastasis is difficult to address using these cells (Francia *et al*., 2011; Prat *et al*., 2010).

## 4 EMT studies in engineered mouse models

To directly address the role of EMT in tumor progression and metastasis, genetic engineered mouse models have been used to determine the presence of EMT in primary tumor cells and to test the requirement of EMT in metastasis *in vivo*. Direct *in vivo* evidence for EMT in metastasis was initially demonstrated in transgenic mouse models where deletion of the E‐cadherin gene led to transition from a well‐differentiated adenoma to an invasive carcinoma in a transgenic mouse model of pancreatic cancer (Perl *et al*., 1998). In metastatic breast cancer models that are largely driven by either PyMT (polyomavirus middle T antigen) or MMTV‐Neu (Erbb2) oncogenes, gene expression profiles of primary tumor have mostly ‘luminal‐like’ or epithelial gene expression signature with only rare cells with mesenchymal phenotype (Herschkowitz *et al*., 2007). Transplanted MMTV‐PyMT tumor cells expressing a mesenchymal marker FSP1 (fibroblast‐specific protein 1) yielded more lung metastases compared with breast cancer cells from PyMT transgenic mice carrying null alleles for FSP1 expression (Xue *et al*., 2003). These findings suggest that tumor cells that have undergone EMT have higher tendency to metastasize.

The role of EMT‐TFs in tumor cell dissemination was supported by a series of studies that label tumor cells expressing EMT‐TFs. Rhim *et al*. observed that tumor cells expressing ZEB1 were found in the invasive tumor cells and in the CTCs of the KPC pancreatic cancer mouse model (Rhim *et al*., 2012). Furthermore, using a yellow fluorescent protein (YFP)‐tagged endogenous SNAIL1 (SNAIL‐YFP), Ye *et al*. showed that SNAIL1 expression is directly correlated with EMT and metastatic potential. As tumors progressed, SNAIL‐YFP‐positive cells gradually acquired basal CK14 expression, lost luminal CK8 expression, expressed elevated levels of EMT‐TFs and mesenchymal markers, and became more aggressive in organoid experiments. When SNAIL1‐YFP‐positive cells (and not SNAIL2‐YFP‐positive cells) isolated from primary tumors were introduced into mice by tail vein injection or subcutaneous implantation (Ye *et al*., 2015), they also formed significantly more metastases.

To directly test the essential role of EMT‐TFs in driving metastasis, Tran *et al*. deleted the SNAIL1 gene specifically in the mammary gland in the MMTV‐PyMT mice and found that SNAIL1 deletion suppressed EMT and drastically blunted lung metastasis (Tran *et al*., 2011). Further supporting the notion that TWIST1 promotes EMT and tumor invasion is the finding that deletion of one allele of TWIST1 gene completely blocked the conversion from benign papilloma to invasive squamous cell carcinoma in the combined and 12‐O‐tetradecanoylphorbol‐13‐acetate (DMBA/TPA) skin tumor model (Beck *et al*., 2015). On the contrary, a recent study by Zheng *et al*. used a pancreatic epithelial cell‐specific PDX1 promoter to delete SNAIL1 or TWIST1 in a KPC (Kras^LSL.G12D/+^; p53^R172H/+^; Pdx1‐Cre) genetic engineered mouse model of pancreatic ductal adenocarcinoma and found that deletion of SNAIL1 or TWIST1 did not lead to any significant difference in metastatic potential and the number of CTCs, but rather an increased sensitivity to gemcitabine treatment (Zheng *et al*., 2015). One likely explanation for the lack of effects on metastasis upon deletion of SNAIL1 or TWIST1 is due to redundant functions of multiple EMT‐TFs in inducing EMT in this KPC tumor model. Specifically, ZEB1 has been shown to be a highly induced EMT‐TF in this tumor model (Rhim *et al*., citation) and could serve as an alternative EMT‐inducing transcription factor to promote metastasis, in addition to SNAIL1 and TWIST1.

One main controversy on the role of EMT in tumor metastasis has been the pathological observation that carcinoma metastasis lesions largely present an epithelial phenotype and morphology and seemingly have not gone through a mesenchymal transition. However, this finding is not contradictory to the EMT hypothesis in metastasis as it has long been postulated that EMT is a reversible program during metastasis by Brabletz *et al*. (2005) and Thiery (2002). They proposed that carcinoma cells turn on the EMT program to invade and disseminate into distant sites, but they will revert into an epithelial state through mesenchymal–epithelial transition (MET) to generate secondary growth in distant organs. Using inducible systems, a few studies have tackled the impact of partial or transient EMT on metastasis.

This first study that provided experimental support for the importance of reversible EMT in metastasis was performed in an inducible TWIST1 mouse model (Tsai *et al*., 2012). Stemming from the observation that endogenous nuclear TWIST1 was found in aggressive human breast tumor samples, but not in distant metastatic lesions, the hypothesis that TWIST1 may be dynamically regulated in primary tumor versus distant metastasis was tested. Using a DMBA‐TPA carcinogen‐induced skin tumor mouse model, Tsai *et al*. demonstrated that induction of TWIST1 at the primary tumor sites potently promoted the conversion of benign papilloma to invasive squamous cell carcinoma. In response to TWIST1 induction, tumor cells underwent EMT, invaded through the basement membrane, and a large number of circulating tumor cells (CTCs) disseminated into the blood circulation. Importantly, continuous induction of TWIST1 in tumor cells that have reached distant organs did not result in increased macrometastases. Instead, turning off TWIST1 expression, which resulted in the reversion of EMT at the distant sites, yielded a large number of metastatic nodules.

Consistent with the importance of reversible EMT in TWIST1‐driven metastasis, endogenous SNAIL1 expression was found to be restricted in cells at the invasive fronts of primary tumors and in CTCs, but not within the macrometastatic lesions in multiple genetic mouse models of breast cancer (Tran *et al*., 2014). By coupling endogenous SNAIL1 reporter with an inducible SNAIL1 transgene, Tran *et al*. showed that transient expression of SNAIL1 is crucial for the formation of macrometastases, while continuous SNAIL1 expression led to a significantly decreased number of lung metastases. Together, independent studies using inducible TWIST1 and SNAIL1 mice support the *in vivo* occurrence of spatiotemporal regulation of EMT‐TFs and their important impact on the metastatic cascade.

Conditional mouse models described above have allowed us to understand the importance of EMT plasticity in the metastatic cascade. Disseminated cancer cells appear to have a tight regulation of expression of EMT‐TF, along with epithelial and mesenchymal genes. However, the inducible mouse models relied on overexpression of EMT‐TFs, and thus, it is possible that such supraphysiological levels of EMT‐TFs could produce unintended additional effects. Another approach to study dynamic EMT in metastasis was to perform lineage tracing experiments in an attempt to reveal whether EMT indeed occurred in tumor cells that eventually resulted in distant metastases. To achieve this goal, the EMT promoter that drives Cre expression needed to be expressed in most if not all of the tumor cells that transiently underwent EMT activation in the primary mammary tumors *in vivo*. More importantly, this EMT promoter needed to be strong enough to activate the reporter gene activation in most if not all tumor cells that transiently underwent EMT. Given the transient and plastic nature of the EMT program, such strong and specific EMT reporter genes are challenging to establish, but have been attempted.

One of the first lineage marking studies by Trimboli *et al*. chose the FSP1 promoter for such purpose because FSP1 has shown to be induced during EMT under some conditions. They developed an FSP1‐Cre transgenic mice in which expression of the Cre recombinase was driven by a 3.1‐kb fragment of the FSP1 promoter (Fsp1‐Cre‐BGH) and crossed this mouse model with three different oncogene‐driven mammary tumor models (WAP‐Myc, MMTV‐PyMT, and MMTV‐Neu) to search for EMT activation in distant lung metastases (Trimboli *et al*., 2008). Surprisingly, FSP1 promoter‐activated X‐gal signal was only observed in a small number of green fluorescent protein (GFP)‐positive cells in the WAP‐Myc mouse model, while no X‐gal signal was observed in the MMTV‐PyMT or MMTV‐Neu mice. The authors concluded that Myc‐driven breast tumors present high EMT characteristics.

Recently, using a different FSP1‐Cre transgenic mouse line, Fischer *et al*. performed similar lineage tracing experiments in MMTV‐PyMT and MMTV‐Neu mice. Interestingly, they were able to observe primary tumor cells that have turned on GFP by the FSP1‐Cre transgene described above. Also, they reported a significant enrichment of GFP‐positive CTCs (presumably cells that have undergone EMT). However, no enrichment of GFP‐positive tumor cells was observed in the resulting distant metastasis lesions (Fischer *et al*., 2015). Therefore, they concluded that EMT was dispensable for metastasis. The different results obtained from these two studies using similar FSP1 promoter to lineage trace cells undergoing EMT in the same PyMT and Neu mouse models suggest that the Cre drivers used for these studies may be responsible for the differential labeling of intended cells.

One additional EMT sensor has been developed using E‐cadherin expression and localization as a reporter to observe EMT in breast tumors of the MMTV‐PyMT mouse model by intravital microscopy (Beerling *et al*., 2016). Using this model, the authors found that tumor cells that lost membrane E‐cadherin and presented with a mesenchymal phenotype, presumably underwent EMT, were capable of migration *in vivo*, while their epithelial counterpart was not. In the circulation, a slightly higher proportion of tumor cells with the loss of E‐cadherin were found. Interestingly, although micrometastases of one to two disseminated tumor cells displayed a partial EMT phenotype, disseminated cell clusters of three or more cells exclusively displayed E‐cadherin, suggesting a phenotypic switch or MET occurring at distant sites to allow macrometastasis formation.

The main challenge of lineage tracing experiments is the reliability of the lineage tracing promoter that drives the Cre. This is especially difficult given the spectral and plastic nature of epithelial and mesenchymal marker expression, which is hard to be captured by the inherently binary lineage marking strategies used to trace cells based on a single gene expression. In particular, the reversible EMT hypothesis suggests that partial, rather than complete, EMT states are more likely to associate with higher aggressiveness, plasticity, and tumor‐initiating properties (Jolly *et al*., 2015). Given the critical role of a partial and plastic EMT in metastasis, further studies using more sensitive EMT reporters are essential to resolve the role of EMT in metastasis.

## 5 Evidence of EMT in human patient tumor samples

Overt evidence of EMT in human tumors has been the most challenging to obtain and interpret; therefore, it is the biggest source for contentious debate on the clinically relevant role of EMT in the metastatic cascade (Ledford, 2011; Tarin *et al*., 2005). One main challenge is that conventional histopathological analysis cannot distinguish between epithelial cancer cells that have undergone EMT and the abundant stromal fibroblasts by morphology or immunohistochemical analyses. To make matters more complicated, the transient reversible EMT process in metastasis renders capturing cells undergoing the transition within human tumor samples difficult.

Only a small number of tumor cells in a single primary carcinoma, with an exception of lobular breast cancer and diffuse gastric carcinoma, present aberrant or loss of E‐cadherin expression (Becker *et al*., 1994; Berx *et al*., 1995). However, numerous clinical studies support the notion that EMT gene signatures predict poor prognosis based on correlation between the expressions of mesenchymal markers such as vimentin, N‐cadherin, fibronectin, and tumor aggressiveness and poor clinical outcome in various cancer tissues by immunohistochemistry or tissue array (Aleskandarany *et al*., 2014; Liu *et al*., 2013; Nitta *et al*., 2014; Sarrió *et al*., 2008; Tsang *et al*., 2013). Certain subtypes of cancer with poor prognosis (i.e., basal‐like, claudin low subtype of breast cancer) have a strong EMT signature (Liu *et al*., 2013; Sarrió *et al*., 2008; Taube *et al*., 2010). A recent meta‐analysis showed a positive correlation between expression of EMT‐TFs (SNAIL1, SNAIL2, and TWIST1) and 3‐year overall survival in 3218 patients with advanced breast cancer (HR 1.72, 95% CI = 1.53–1.93) (Imani *et al*., 2016). Besides in breast cancer, expression levels of TWIST1 and SNAIL are also associated with poor prognosis in lung, gastrointestinal, hepatobiliary, head‐and‐neck, and urinary cancers as demonstrated in a meta‐analysis of 4938 patients in 38 studies showing poorer overall survival in those with high expression of TWIST1 or SNAIL1 (HR = 2.07 with 95% CI 1.63–2.63 and HR = 1.63 with 95% CI 1.33–1.99, respectively) (Zhang *et al*., 2014). EMT‐TFs, such as TWIST1, are also found to be highly expressed in a subpopulation of cells within a tumor with the highest invasive and metastatic potential at the invading front of the primary tumor, further supporting the importance of EMT in the metastatic cascade (Celesti *et al*., 2013). Finally, expression of EMT‐TFs has been correlated negatively with pathologic response to treatment, suggesting a role in chemoresistance (Creighton *et al*., 2009; Farmer *et al*., 2009).

The most direct evidence for a role of EMT in metastasis comes from analyses of CTCs in patients with cancer. Analysis of CTCs allows studying human tumor cells that have gained migratory and invasive properties. The presence of CTCs in breast, prostate, colorectal, pancreatic, and lung cancer patients is a poor prognostic factor, supporting the role and requirement for tumor cell invasion and dissemination for metastatic formation and poor clinical outcome (Cristofanilli *et al*., 2004; Goldkorn *et al*., 2014; Han *et al*., 2014; Huang *et al*., 2013; Janni *et al*., 2016; Lucci *et al*., 2012; Rahbari *et al*., 2010). As discussed above, the EMT plasticity in metastasis was indeed reflected in CTCs both in mouse tumor models and in human patients (Celià‐Terrassa *et al*., 2012; Tsai *et al*., 2012). Co‐expression of epithelial and mesenchymal genes was commonly found in CTCs from breast, prostate, colon, gastric, liver, and lung cancer patients, while the corresponding primary tumors rarely simultaneously express both types of markers (Armstrong *et al*., 2011; Wu *et al*., 2015; Yu *et al*., 2013). In addition, a higher proportion of CTCs expressing TWIST1, SNAIL1, or vimentin were identified in patients with advanced stage cancer compared to those with early‐stage cancer, suggesting that these cells may prevail during disease progression and contribute to metastatic outgrowth (Aktas *et al*., 2009; Barrière *et al*., 2012; Giordano *et al*., 2012; Kallergi *et al*., 2011; Papadaki *et al*., 2014; Wu *et al*., 2015). In fact, the presence of CTCs undergoing EMT as measured by the overexpression of TWIST1 or SNAIL1 by qRT‐PCR was predictive of early disease relapse (Mego *et al*., 2012). In patients with hepatocellular carcinoma, acquisition of EMT phenotype in CTCs, such as TWIST1 and vimentin expression, was associated with disease progression and increased metastatic formation (Li *et al*., 2013). Furthermore, the presence of mesenchymal CTCs was correlated with a higher risk of treatment failure in breast, lung, and colorectal cancer (Kallergi *et al*., 2011; Satelli *et al*., 2014; Togo *et al*., 2014; Yu *et al*., 2013). Using a multiplex tumor and EMT dual colorimetric RNA *in situ* hybridization analysis, Yu *et al*. elegantly demonstrated that CTCs were more enriched with a mesenchymal phenotype change upon treatment and correlated with poor clinical outcomes (Yu *et al*., 2013). In summary, CTCs represent the dynamic EMT state that tumor cells adapt within the circulation and are closely connected to the potential of metastasis and poor prognosis. Further molecular characterization of CTCs using global gene expression profiling in large patient cohorts may allow a more complete assessment of the CTC‐EMT signature with regard to prognostic potential and treatment prediction.

## 6 Targeting EMT: therapeutic implications in treatment for metastatic cancer

As discussed above, research in the past decade supports an important role of EMT in facilitating tumor metastasis, and novel therapies targeting the residual EMT‐driven cancer cells in combination with conventional therapy may decrease metastasis formation and drug resistance. To this end, preclinical studies have tested a number of small‐molecule inhibitors that target stimuli or signaling pathways associated with EMT, which are summarized in a recent review (Marcucci *et al*., 2016). Based on our current understanding of the molecular and cellular plasticity of the EMT program, treatment strategies using anti‐EMT therapies should be carefully considered and tested. While preventing EMT may be beneficial in the treatment of epithelial cancer cells that are prone to acquire mesenchymal traits, reversion of EMT may also have unintended consequence of promoting regrowth of already disseminated tumor cells. Conceptually, eliminating mesenchymal tumor cells or inhibiting their growth in combination with conventional therapies that target the epithelial population may be most suitable to address the dynamic partial EMT state and tumor heterogeneity. Additional biomarkers, such as a transcriptomic EMT signature (Tan *et al*., 2014) from primary tumors or circulating tumor cells., will also aid in identifying appropriate patients and therapeutic window in which anti‐EMT therapy could yield better treatment responses and improve survival.

## 7 Conclusion

The complex nature of EMT in metastasis requires careful analysis to dissect the dynamic cellular plasticity. Intense research in the past two decades on EMT has converged into a common theme that EMT indeed occurs *in vivo* during tumor progression and CTCs are highly enriched with an EMT gene signature. Various studies in cancer cell lines, mouse tumor models, and human tumor samples strongly support the notion that EMT is a key mechanism for effective metastatic dissemination. It is also clear that a partial and reversible EMT is important to allow disseminated tumor cells to revert to an epithelial phenotype for metastatic outgrowth in distant sites as illustrated in Fig. 1. A few recent studies failed to detect EMT in disseminating tumor cells using certain EMT reporters, and it therefore raises the possibility that certain carcinoma cells could disseminate and form distant metastases without undergoing EMT. Given the difficulty to sensitively detect activation of the transient and partial EMT program *in vivo*, further studies are needed to develop more efficient EMT reporters to further evaluate the involvement of EMT in metastasis to clearly settle this decade‐old debate. The potential of EMT gene signature in CTCs to predict survival and treatment response needs to be further evaluated for clinical applications. Development of modulators of EMT may prove to be a useful research tool to further dissect the regulatory pathways involved in EMT and metastasis, in addition to preventing or slowing metastatic outgrowth in the clinical setting.
